# Large deletion in *PIGL*: a common mutational mechanism in CHIME syndrome?

**DOI:** 10.1590/1678-4685-GMB-2017-0172

**Published:** 2018-02-19

**Authors:** José RM Ceroni, Guilherme L Yamamoto, Rachel S Honjo, Chong A Kim, Maria R Passos-Bueno, Débora R Bertola

**Affiliations:** 1 Genetics Unit, Instituto da Criança do Hospital das Clínicas, Faculdade de Medicina, Universidade de São Paulo, São Paulo, SP, Brazil.; 2 Centro de Pesquisa sobre o Genoma Humano e Células-Tronco, Instituto de Biociências, Universidade de São Paulo, São Paulo, SP, Brazil.

**Keywords:** PIGL, CHIME syndrome, GPI biosynthesis, large deletion

## Abstract

CHIME syndrome is an extremely rare autosomal recessive multisystemic disorder caused by mutations in *PIGL*. PIGL is an endoplasmic reticulum localized enzyme that catalyzes the second step of glycosylphosphatidylinositol (GPI) biosynthesis, which plays a role in the anchorage of cell-surface proteins including receptors, enzymes, and adhesion molecules. Germline mutations in other members of GPI and Post GPI Attachment to Proteins (PGAP) family genes have been described and constitute a group of diseases within the congenital disorders of glycosylation. Patients in this group often present alkaline phosphatase serum levels abnormalities and neurological symptoms. We report a CHIME syndrome patient who harbors a missense mutation c.500T > C (p.Leu167Pro) and a large deletion involving the 5’ untranslated region and part of exon 1 of *PIGL*. In CHIME syndrome, a recurrent missense mutation c.500T > C (p.Leu167Pro) is found in the majority of patients, associated with a null mutation in the other allele, including an overrepresentation of large deletions. The latter are not detected by the standard analysis in sequencing techniques, including next-generation sequencing. Thus, in individuals with a clinical diagnosis of CHIME syndrome in which only one mutation is found, an active search for a large deletion should be sought.

CHIME syndrome (OMIM #280000) is an extremely rare autosomal recessive multisystemic disorder, originally described in 1983 [Zunich and Kaye, 1983], who proposed the discovery of a new neuroectodermal syndrome. The acronym CHIME (colobomas, congenital heart defect, migratory ichthyosiform dermatosis, mental retardation, and ear anomalies) was proposed (Shashi *et al.*, 1995) based on the presence of the most consistent clinical manifestations. CHIME syndrome is caused by biallelic mutations in *PIGL* (Ng *et al.*, 2012). PIGL is an endoplasmic reticulum localized enzyme that catalyzes the second step of glycosylphosphatidylinositol (GPI) biosynthesis, which plays a role in the anchorage of more than 150 cell-surface proteins including receptors, enzymes and adhesion molecules. Germline mutations in other members of PIG family genes, as well as members of Post GPI Attachment to Proteins (PGAP), involved in structural remodeling of GPI after its attachment to proteins, have been described on a recently emerging group of diseases within congenital disorders of glycosylation. The phenotypic spectrum of these disorders is still not completely known, but clinical overlap between the majority of the disorders in this group is recognized, as patients often present alkaline phosphatase (ALP – which is a GPI-anchored protein) serum levels abnormalities and neurological symptoms, including seizures, hypotonia and mental retardation (Belet *et al.*, 2014). To date, eight probands with mutations in *PIGL* have been reported, six of them with the diagnosis of CHIME syndrome (Ng *et al.*, 2012, Knight Johnson *et al.*, 2017), and two of them presenting clinical findings compatible with Mabry syndrome, characterized by mental retardation and hyperphosphatasia (Fujiwara *et al.*, 2015, Pagnamenta *et al.*, 2017). A recurrent missense mutation c.500T > C (p.Leu167Pro) has been found in heterozygosity in all patients with CHIME syndrome, associated with a null mutation in the other allele, including here two large deletions of different sizes. In contrast, in the two patients with the diagnosis of Mabry, two null mutations were retrieved in *PIGL*.

We report on an individual presenting a full-blown phenotype of CHIME syndrome that harbors, besides the common missense mutation in one allele, a large deletion involving the 5’ untranslated region and part of exon 1 of *PIGL* in the opposite allele. This report gives further support to the fact that large deletions could be a common type of mutations in the second allele in individuals with CHIME syndrome.

The proband is a 4 year-old boy, the first and only child of non-consanguineous parents. During the pregnancy, fetal ultrasound disclosed unilateral hydronephrosis. He was born preterm (35 w), with a birth weight of 3150 g, length of 48 cm and Apgar scores of 9/10. He evolved with respiratory distress and sepsis, requiring hospitalization for 21 days. His developmental milestones were delayed: he sat unsupported by 10 months of age, walked at 22 months of age, and he has no speech at the age of 4. He has epilepsy, with a first seizure at 8 months of age during a febrile episode. A partial control of the seizures was obtained with the use of two anti-epileptic drugs. The parents stated that his skin has always been dry and he developed hyperpigmented/ichthyosiform lesions at the age of 1 year. Physical examination at 1 year 9/12 showed: weight of 16 Kg (p > 95^th^ centile), Height of 94 cm (p > 95^th^ centile), OFC of 52 cm (p > 98^th^ centile); fine hair, which is sparse in the temporal regions, prominent forehead, ocular hypertelorism, upslanting palpebral fissures, everted lower lip, small, widely-spaced teeth, with fusion of the central and lateral right incisors, and uplifted ear lobes; short neck; large hands, with brachydactyly, finger pads and incomplete single palmar creases; hyperchromic/ichthyosiform skin lesions in neck, axillas and knees, geographic lesions with hyperpigmented borders in the thorax and abdominal areas and palmar hyperkeratosis (Figure 1). Complementary exams disclosed: normal echocardiography and cranial CT scan; hand X-rays with short distal phalanges of all fingers, as well, as the medial phalanx of the left fifth digit, and advanced bone age; ophthalmologic evaluation, bilateral retinal coloboma; renal ultrasound, bilateral pyelocaliceal ectasia; alkaline phosphatase (ALP) serum levels of 456 U/L and 319 U/L (normal range 150-380 U/L); pyridoxal-phosphate 27.2 ug/L (normal range 5.2-34 ug/L); normal blood and skin G-banded karyotype and chromosomal microarray. No formal audiologic evaluation was performed. As the patient showed clinical features compatible with the diagnosis of CHIME syndrome, whole exome sequencing (WES) was performed and the recurrent heterozygous mutation p.Leu167Pro in *PIGL* was identified. In order to seek for the second mutation, we analyzed this gene with the Integrative Genomics Viewer (IGV) and a deletion of 802 base pairs ([hg19] chr7: 16,119,889-16,120,690) involving the 5’ untranslated region and the first 50 aminoacids, including the ATG start codon in exon 1 was observed (Figure 2). This gene alteration was confirmed by bridging PCR and Sanger sequencing (Fig. 1). Clinical and molecular data of the present patient, as well as the other individuals reported in literature with mutations in *PIGL* are shown in Table 1 (Zunich and Kaye, 1983, Shashi *et al.*, 1995, Tinschert *et al.*, 1996, Schnur *et al.*, 1997, Sidbury and Paller, 2008, Ng. *et al.*, 2012, Fujiwara *et al.*, 2015, Knight Johnson *et al.*, 2017, Pagnamenta *et al.*, 2017).

**Figure 1:**
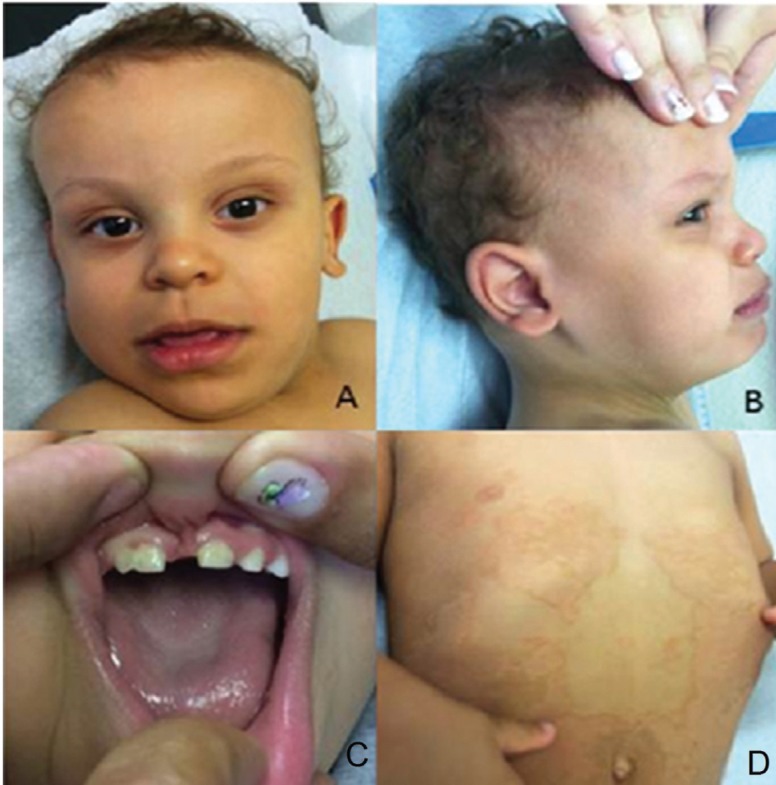
A-D. Fine, sparse hair in the temporal regions, proeminent forehead, ocular hypertelorism, upslanting palpebral fissures, everted lower lip, small, widely-spaced teeth, diastema and fusion of the central and lateral right inceisors, uplifted ear lobes; short neck; hyperchromic/icthyosiform skin lesions, geographic lesions with hyperpigmented borders in the thorax and abdominal areas.

**Figure 2:**
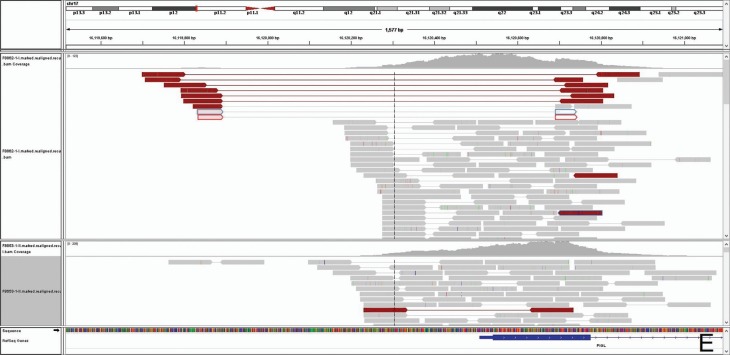
IGV analysis of *PIGL* showing a deletion of 802 base pairs ([hg19] chr7: 16,119,889-16,120,690) involving the 5’ untranslated region and the first 50 aminoacids, including the ATG start codon in exon 1.

**Table 1:** Clinical and molecular findings of patients with *PIGL* mutations

Clinical Findings	Zunich and Kaye, 1983; Shashi *et al.*, 1995; Ng. *et al.*, 2012		Shashi *et al.*, 1995; Ng. *et al.*, 2012	Tinschert *et al.*, 1996; Ng. *et al.*, 2012	Schnur *et al.*, 1997; Ng. *et al.*, 2012	Sidbury and Paller, 2008; Ng. *et al.*, 2012	Fujiwara *et al.*, 2015	Knight Johnson *et al.*, 2017	Pagnamenta *et al.*, 2017	Our patient	Total
	Patient 1	Patient 2 (brother of patient 1)	Patient 3	Patient 4	Patient 5	Patient 6	Patient 7	Patient 8	Patient 9	Patient 10	10 (9 families)
**Age at initial report**	3y	< 1y	2y	21m	3y	10y	< 1y	3y	NA	4y	-
**Age of last evaluation**	16y	7y	15.5y	21m	Died at 5y	10y	3y	3y	NA	6y	-
**Sex**	M	M	F	F	F	M	F	M	F	M	5M:5F
**Ethnicity / origin**	Irish-Dutch / Polish		NA	NA	Caucasian	NA	Japanese	Caucasian	Caucasian	Brazilian	
**Prenatal ultrasonographic findings**								CLP, Cardiac (DOV, PPS)		HN (unilateral)	3/9 (33.3%)
**Neonatal**											
Gestational age	Term	Term	Term	37w	42w	NA	33w	Term	39w	35w	Term 7/9 (77.7%)
Birth weight (centile)	4Kg 95^th^	3.72Kg (90^th^	3.5Kg 75^th^	3.18Kg 95^th^	4.15Kg 95^th^	NA	2.51Kg 90^th^	4.4Kg 98^th^	4.37Kg 90^th^	3.15Kg 90^th^	BW > 90% (8/9=88.8%)
Birth length (centile)	53.5cm 95^th^	53cm > 90^th^	52cm 95^th^	48cm 95^th^	57cm 95^th^	NA	51cm > 95^th^	NA	NA	48cm 75^th^	BL > 90%/(6/7=85.7%)
Complications			Respiratory distress	hypothermia, hypocalce mia, hypoglice mia	Transient tachycardia, transient hypoglicemia				NA	Respiratory distress and sepsis	
**Growth (age)**	16y	7y	14.5y	21m	3.5y	NA	1y10m	NA	NA	3y10m	
Weight (centile)	61Kg (25^th^)	19.5Kg (< 5^th^)	39Kg (5^th^)	NA	19.86Kg (> 95^th^)	NA	7.9Kg (< 3^rd^)	NA	(50-75^th^)	25 (> 95^th^)	W > 90% 2/7
Height (centile)	172cm (25^th^)	109cm (< 5^th^)	152cm (10^th^)	81cm (10th)	104cm (80^th^)	NA	80cm (5^th^)	NA	(50-75^th^)	107 (90^th^)	H > 90% 1/8
OFC (centile)	NA	NA	45cm (< 5^th^)	45cm (10th)	50cm (60^th^)	NA	NA	NA	(50-75^th^)	54 (> 98^th^)	OFC > 90% 1/5
**Craniofacial**											
Sparse, fine hair	+	+	+	+	+	-	-	+	NA	+	7/9 (77.7%)
Brachycephaly	+	+	+	+	+	-	-	NA	NA	-	5/8 (62.5%)
Hyperthelorism	+	+	+	+	+	-	+	+	NA	+	8/9 (88.8%)
Retinal coloboma	+	+	+	+	+	+	-	+	NA	+	8/9 (88.8%)
Flat/broad nasal root	+	+	+	+	+	+	-	+	NA	-	7/9 (77.7%)
Short philtrum	+	-	-	+	-	-	-	+	NA	-	3/9 (33.3%)
Wide mouth	+	-	+	+	+	-	-	+	+	-	6/10 (60%)
Full lips	+	+	+	+	-	-	-	-	NA	-	4/9 (44.4%)
Cleft palate	+	+	-	-	-	-	-	+	-	-	3/10 (30%)
Widely spaced teeth	+	+	+	-	+	-	-	-	+	-	4/10(40%)
Overfolded helices	+	+	+	+	+	-	+	+	NA	+	8/9 (88.8%)
**Conductive deafness**	+	+	+	+	+	+	+	NA	+	NA	8/8 (100%)
**Cardiac anomaly**	-	TGA	VSD	VSD	-	SAS	-	DOV/PPS	NA	-	5/9 (55.5%)
**Skeletal**											
Clinodactyly	-	-	+	-	+	+	-	NA	+	-	4/9 44.4%)
Brachydactyly	+	-	-	+	-	+	-	NA	+	+	5/9 (55.5%)
Broad second toe	+	+	+	+	-	-	-	NA	NA	-	4/8 (50%)
**Central nervous system**											
Developmental delay/ Intellectual disability	+	+	+	+	+	+	+	+	+	+	10/10 (100%)
Seizures	+	+	+	+	+	+	+	+	+	+	10/10 (100%)
Wide based gait	+	+	+	+	+	+	NA	NA	NA	+	7/7 (100%)
Cerebral atrophy	+	-	-	+	+	NA	+	NA	-	-	4/8 (50%)
**Renal**	NA	NA	UJO	ERP	HN	HN	-	NA	RC	UJO	6/7 (85.7%)
**Skin**											
Icthyosiform rash	+	+	+	+	+	+	-	+	-	+	8/10 (80%)
Thick palms/soles	+	+	+	+	-	-	-	NA	-	+	5/9 (55.5%)
**ALP serum level**	NA	NA	NA	NA	Mildy elevated (NA)	NA	4,394 IU/L (NR 395-1,289 IU/L)	NA	575U/l; 923 U/l; 819 U/l (NR 100–400 U/l)	456U/L and 319U/L (NR 150-380U/L)	Elevated ALP 4/4 (100%)
**Others**	sinus infections	Violent behaviour	Hip subluxation; middle ear infections, violent behaviour	Clubfoot, lipoma	ALL (4y)	NA			Pectus excavatum, bro ad hallux, cutis marmorata, dry skin, one strawberry naevus		
*PIGL* mutations	p.Leu167Pro/p.Leu92Phefs* 15		p.Leu167Pro/p.Gln218*	p.Leu167Pro/del17p12-p11. 2 (array-CGH)	p.Leu167Pro/?	p.Leu167Pro/C.427-1G > A	p.Leu13Alafs* 11/p.Glu86Aspfs* 2	p.Leu167Pro/c.426+6654_660 +3131del	p.Trp16*/p.Asp113fs*2	p.Leu167Pro/802 base pairs del ([hg19] chr7: 16,119,889-16, 120,690)	

NA=not available; M=male; F=female; TGA=transposition of the great arteries; PPS=peripheral pulmonic stenosis; SAS=subaortic stenosis; VSD=ventricular septal defect; DOV=double outlet ventricule; UJO=uteropelvic junction obstruction; ERP=unilateral ectopic renal pelvis; HN=hydronephrosis; RC=renal cysts; ALL=acute limphobastic leukemia.

*PIGL* mutations have been reported mainly in individuals with CHIME syndrome (eight individuals, including a pair of brothers and the present case), one individual reported by Fujiwara (Fujiwara *et al.*, 2015), clinically diagnosed as Mabry syndrome, and one individual recently reported by Pagnamenta (Pagnamenta *et al.*, 2017) whose clinical and laboratorial findings were also reminiscent of Mabry syndrome. In the eight individuals with CHIME syndrome with *PIGL* proven mutations (Ng *et al.*, 2012, Fujiwara *et al.*, 2015, Knight Johnson *et al.*, 2017, Pagnamenta *et al.*, 2017), we could retrieve a homogeneous phenotype, characterized by developmental delay/intellectual disability, epilepsy, ocular coloboma, conductive hearing loss and icthyosiform skin rash in all of them. In the neonatal period, some malformations could be observed by fetal ultrasssonography in a few patients (3/9), such as cardiac abnormality, cleft lip/palate, and hydronephrosis. The majority of the individuals were born at term, with high birth weight and length. The most frequent complications found in the neonatal period were respiratory distress (2/9) and hypoglicemia (2/9). Nevertheless, overgrowth was not frequently observed in the evolution: only two individuals presented with weight and/or height greater than 2SD above the mean. Facial dysmorphisms were represented mainly by sparse, fine hair, ocular hypertelorism, flat/broad nasal root, ears with overfolded helices. Different structural cardiac abnormalities were observed in 5/9, including isolated VSD, valvar stenosis and outflow tract defects, as well as renal anomalies (6/7), mainly hydronephrosis. Clinodactyly/brachydactyly and thickness of the palms and soles could be observed in approximately 50% of the cases. One individual developed acute lymphoblastic leukemia (ALL) at the age of 4. This is the only malignant neoplastic condition described in CHIME individuals. As this hematological abnormality is common in the pediatric population, we cannot exclude that this association could be fortuitous. ALP serum levels have not been routinely measured in patients harboring *PIGL* mutations, with only one patient reported as presenting mild elevation (Schnur *et al.*, 1997). The present patient also showed mild elevations on two different occasions. The degree of abnormality in the ALP serum levels is partly explained by the position of the enzyme in the GPI biosynthesis pathway: proteins responsible for the latter steps are prone to lead to higher ALP serum levels, as this is seen in Mabry syndrome, mainly caused by mutations in *PIGV* (Krawitz *et al.*, 2010, Murakami *et al.*, 2012). In contrast, *PIGL*, responsible for the second step, lead to mild elevations (Murakami *et al.*, 2012). However, high ALP serum levels were retrieved in two individuals harboring biallelic null mutations (frameshift and/or nonsense) in *PIGL* (Fujiwara *et al.*, 2015, Pagnamenta *et al.*, 2017), indicating that, not only the physical localization of these different enzymes in the GPI biosynthesis pathway are important to determine the secretion of GPI-anchored proteins, but also their residual functional activity.

In all the eight probands described so far with CHIME syndrome, in which the molecular analysis was performed, the missense mutation c.500T > C (p.Leu167Pro) was present in one allele, combined with a null mutation in the opposite allele: frameshift mutation (1), nonsense mutation (1), splicing mutation (1), and large deletions (3). In one individual, the authors failed to identify the second mutation. Although the number of patients with proven mutations in *PIGL* is too small to draw definitive conclusions, we could observe an overrepresentation of large deletions, which sizes ranged from 802bp, as the one found in the present patient, to 1Mb deletion in 17p12-11.2, encompassing the whole gene (Tinschert *et al.*, 1996). In the patient reported by Knight Johnson (Knight Johson *et al.*, 2017), the breakpoints of the deletion involving *PIGL* exons 4 to 6 occurred within Alu-repeats. As these Alu sequences were overrepresented in the intronic regions of *PIGL*, the authors suggested that copy number variations could occur as a consequence of this specific genomic architecture. The breakpoints of the deletion presented by our patient do not follow this rule, as one of them occurs within exon 1. Large deletions are not detected by the standard analysis of sequencing techniques, including next-generation sequencing. This could be the case in the patient reported by Ng (Ng *et al.*, 2012) in which only one mutation was identified. Thus, in individuals with a clinical diagnosis of CHIME syndrome in which only one mutation is found, especially the recurrent p.Leu167Pro, an active search for a large deletion should be sought.

The whole phenotypic and genotypic spectrum in individuals harboring mutations in *PIGL* is still incompletely characterized. Interestingly, individuals presenting two null mutations in *PIGL* did not present the typical CHIME syndrome phenotype. Instead, their phenotype was compatible with the diagnosis of Mabry syndrome, with severe neurological involvement and high serum levels of APL. Thus, the residual protein function coded by *PIGL* is important in the phenotypic delineation. It remains to be determinated if the presence of two less severe mutations, such as, two missense mutations, would lead to a mild phenotype or even lack of phenotypic expression. Further reports are required to clarify this matter.
